# Laboratory-Confirmed COVID-19 Case Incidence Rates Among Residents in Nursing Homes by Up-to-Date Vaccination Status — United States, October 10, 2022–January 8, 2023

**DOI:** 10.15585/mmwr.mm7204a3

**Published:** 2023-01-27

**Authors:** Heather Dubendris, Hannah E. Reses, Emily Wong, Phillip Dollard, Minn Soe, Meng Lu, Jonathan R. Edwards, Tamara Pilishvili, Theresa Rowe, Andrea Benin, Jeneita M. Bell

**Affiliations:** ^1^Division of Healthcare Quality Promotion, National Center for Emerging and Zoonotic Infectious Diseases, CDC; ^2^Lantana Consulting Group, East Thetford, Vermont; ^3^Division of Viral Diseases, National Center for Immunization and Respiratory Diseases, CDC.

Nursing home residents have been disproportionately affected by COVID-19; older age, comorbidities, and the congregate nature of nursing homes place residents at higher risk for infection and severe COVID-19–associated outcomes, including death (*1*). Studies have demonstrated that receipt of a primary COVID-19 mRNA vaccination series (*2*) and monovalent booster doses (*3*) is effective in reducing COVID-19–related morbidity and mortality in this population. Public health recommendations for staying up to date with COVID-19 vaccination have been revised throughout the pandemic response, most recently to include an updated (bivalent) booster dose, which protects against both the ancestral strain of SARS-CoV-2 and recent Omicron variants BA.4 and BA.5 (*4*). However, data on the effectiveness of staying up to date, including with bivalent booster doses, are lacking among nursing home residents. CDC’s National Healthcare Safety Network (NHSN) analyzed surveillance data to examine weekly incidence rates of COVID-19 among nursing home residents by up-to-date vaccination status (receipt of a bivalent booster dose or completion of a primary series or receipt of a monovalent booster dose within the previous 2 months [i.e., not yet eligible to receive a bivalent booster dose]).* Up-to-date vaccination status among nursing home residents remained low throughout the study period, increasing to 48.9% by the week ending January 8, 2023. During October 10, 2022–January 8, 2023, the COVID-19 weekly incidence rates (new cases per 1,000 nursing home residents) among residents who were not up to date with COVID-19 vaccination were consistently higher than those among residents who were up to date. Moreover, the weekly incidence rate ratios (IRRs) indicated that residents who were not up to date with COVID-19 vaccines had a higher risk for acquiring SARS-CoV-2 than their up-to-date counterparts (IRR range = 1.3–1.5). It is critical that nursing home residents stay up to date with COVID-19 vaccines and receive a bivalent booster dose to maximize protection against COVID-19.

Nursing homes began reporting numbers of laboratory-confirmed COVID-19 cases (a newly positive SARS-CoV-2 viral test result received by a resident) and vaccination data to NHSN in April 2020 and December 2020, respectively, and federal mandates issued by the Centers for Medicare & Medicaid Services (CMS) require CMS-certified nursing homes to report these data weekly.^†^^,^^§^ The method for collecting laboratory-confirmed COVID-19 case data in nursing homes has been described (*2*). Vaccination data collection includes the weekly number of residents in the nursing home (with a stay of ≥24 hours) stratified by vaccination status, including the number of residents who are up to date with recommended COVID-19 vaccination.

NHSN analyzed weekly COVID-19 case and vaccination status data during October 10, 2022–January 8, 2023, for CMS-certified nursing homes to assess the data collected based on NHSN’s 2022 fourth quarter (October–December) definition of up-to-date COVID-19 vaccination status.^¶^ The study paired weekly incident case counts by vaccination status with weekly resident counts by vaccination status for each nursing home to calculate crude COVID-19 incidence rates with 95% CIs, by up-to-date vaccination status for each reporting week. Case counts were combined with resident vaccination counts from 2 weeks earlier, because COVID-19 case vaccination status is classified according to vaccination status 14 days before receipt of a positive SARS-CoV-2 test result.

Facilities with missing case or vaccination data were excluded from the analysis. NHSN calculated IRRs by up-to-date vaccination status each week. NHSN also analyzed a subset of data from facilities voluntarily reporting dates, types, and number of primary series doses and booster doses received by each resident (rather than weekly aggregate totals) to calculate the proportion of up-to-date residents who had received a bivalent booster dose.** Analyses were performed using SAS software (version 9.4; SAS Institute). This activity was reviewed by CDC and was conducted consistent with applicable federal law and CDC policy.^††^

Among 16,352 nursing homes, 15,049 (92%) in the 50 U.S. states and the District of Columbia reported case and vaccination data for ≥1 week during the 13-week study period.^§§^ After exclusions, 192,289 facility-weeks (97%) were included in the analysis. An average of 14,791 facilities reported both COVID-19 vaccination and case data each week (range = 14,622–14,874). Facility size (the number of health care personnel per facility) varied, with a median of 116 health care personnel per facility (IQR = 82–165) (Figure). The percentage of residents with up-to-date COVID-19 vaccination status increased slightly during the study period, beginning the week of October 23, from 37.5% to 48.9%; this incremental increase was similar across all facility sizes and geographic regions.

**FIGURE:**
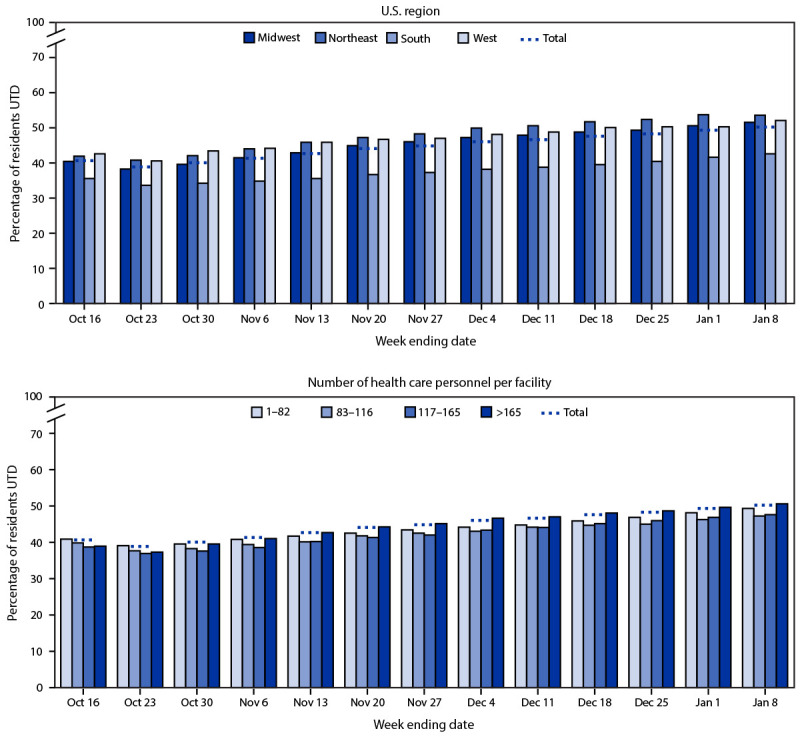
Percentage of nursing home residents with up-to-date COVID-19 vaccination,* by U.S. region, number of health care personnel per facility,^†^ and week^§^ — United States, October 10, 2022–January 8, 2023 **Abbreviation**: UTD = up to date with COVID-19 vaccines. * Receipt of a bivalent booster dose or completion of a primary series or receipt of a monovalent booster dose within the previous 2 months (i.e., not yet eligible to receive a bivalent booster dose). ^†^ Used as an estimate of facility size. ^§^ For each reporting week, vaccination data from 2 weeks earlier are included to allow for appropriate incidence rate calculation.

Each week, COVID-19 incidence rates among nursing home residents who were not up to date with COVID-19 vaccination were higher than those among residents who were up to date (Table). Incidence rates among residents who were up to date with COVID-19 vaccination ranged from 7.2 per 1,000 residents (week ending November 13, 2022) to 15.6 (week ending January 8, 2023) during this period, while incidence rates among those who were not up to date ranged from 9.5 (week ending October 16, 2022) to 18.8 (weeks ending December 11, 2022, and January 8, 2023). IRRs between residents who were not up to date with COVID-19 vaccination and those who were up to date were statistically significant and ranged from 1.3 to 1.5. Among the 15,049 nursing homes included in this study, 1,759 (11.7%) voluntarily reported additional details on vaccine doses for each resident rather than weekly aggregate totals. Analysis of these data for each week of the study period indicated that >99% of residents classified as being up to date had received a bivalent booster dose.

**TABLE:** Weekly* crude COVID-19 incidence rate^†^ among nursing home residents, by up-to-date COVID-19 vaccination status^§^ and incidence rate ratios between those not up to date and those up to date — United States, October 10, 2022–January 8, 2023

Week ending date	UTD status						Incidence rate ratio (95% CI)
	UTD			Not UTD			
	Total residents	No. of cases	Incidence rate (95% CI)	Total residents	No. of cases	Incidence rate (95% CI)	
**2022**							
Oct 16	**454,826**	3,587	7.5 (7.3–7.8)	**737,238**	7,006	9.5 (9.3–9.7)	1.3 (1.2–1.3)
Oct 23	**469,700**	3,396	7.5 (7.2–7.7)	**757,540**	7,582	10.0 (9.8–10.2)	1.3 (1.3–1.4)
Oct 30	**487,216**	4,042	8.6 (8.3–8.9)	**742,733**	8,378	11.3 (11.0–11.5)	1.3 (1.3–1.4)
Nov 6	**500,640**	3,991	8.2 (7.9–8.4)	**730,645**	7,850	10.7 (10.5–11.0)	1.3 (1.3–1.4)
Nov 13	**521,360**	3,611	7.2 (7.0–7.5)	**710,034**	7,658	10.8 (10.5–11.0)	1.5 (1.4–1.6)
Nov 20	**529,696**	4,003	7.6 (7.4–7.9)	**699,221**	7,802	11.2 (10.8–11.3)	1.5 (1.4–1.5)
Nov 27	**545,358**	5,060	9.6 (9.3–9.8)	**687,104**	9,346	13.6 (13.3–13.9)	1.4 (1.4–1.5)
Dec 4	**549,207**	6,708	12.3 (12.0–12.6)	**675,346**	12,227	18.1 (17.8–18.4)	1.5 (1.4–1.5)
Dec 11	**560,306**	7,680	14.0 (13.7–14.3)	**662,469**	12,433	18.8 (18.4–19.1)	1.3 (1.3–1.4)
Dec 18	**570,283**	7,302	13 (12.7–13.3)	**650,398**	11,429	17.6 (17.3–17.9)	1.3 (1.3–1.4)
Dec 25	**580,777**	7,179	12.6 (12.3–12.9)	**644,824**	10,153	15.7 (15.4–16.1)	1.3 (1.2–1.3)
**2023**							
Jan 1	**586,834**	8,261	14.2 (13.9–14.5)	**629,796**	11,280	17.9 (17.6–18.2)	1.3 (1.2–1.3)
Jan 8	**454,826**	9,157	15.6 (15.3–15.9)	**613,280**	11,536	18.8 (18.5–19.2)	1.2 (1.2–1.2)

**Abbreviation:** UTD = up to date with COVID-19 vaccines.

* For each reporting week, total residents by vaccination status from 2 weeks earlier are included to allow for appropriate incidence rate calculation.

^†^ New cases per 1,000 nursing home residents.

^§^ Receipt of a bivalent booster dose or completion of a primary series or receipt of a monovalent booster dose within the previous 2 months (i.e., not yet eligible to receive a bivalent booster dose).

^¶^ Incidence rate ratios are calculated as the incidence rate among residents who were not UTD divided by the incidence rate among residents who were UTD; 95% CIs calculated using Mid-P.

## Discussion

Weekly incidence rates of COVID-19 among nursing home residents who were not up to date with COVID-19 vaccines were 30%–50% higher than were those among residents who were up to date during October 10, 2022–January 8, 2023. Among the subset of nursing homes reporting additional details on vaccine doses for each resident, almost all residents with up-to-date COVID-19 vaccination status had received a bivalent booster dose, suggesting that up-to-date vaccination status can be used to represent bivalent booster dose coverage among nursing home residents. The findings in this report are consistent with other recent studies supporting effectiveness of bivalent booster doses, including a study among adults aged ≥18 years demonstrating that bivalent booster doses maximized protection against symptomatic SARS-CoV-2 infection compared with protection from monovalent vaccination alone (*5*). Another recent study found that bivalent booster doses produced a robust immunologic response in nursing home residents (*6*). Bivalent booster doses have also been shown to provide additional protection against severe outcomes associated with COVID-19, compared with monovalent vaccination alone, including protection against COVID-19–associated emergency department and urgent care visits among adults aged ≥18 years and protection against COVID-19 hospitalization among adults aged ≥65 years (*7*,*8*). Public health efforts to sustain up-to-date COVID-19 vaccination status among nursing home residents (including recommended vaccinations and booster doses) are critical to protecting this population.

Although bivalent booster doses were recommended during fall 2022 (*4*), and the time to receive the bivalent booster dose and remain up to date according to current recommendations has been limited, the proportion of nursing home residents in this study who were up to date (48.9%) was lower than the percentage of nursing home residents who completed a primary series (86.1%) and who received monovalent booster doses (87.0%).^¶¶^ Bivalent booster doses are recommended for nursing home residents who previously received monovalent doses to stay up to date. There might be several reasons that nursing home residents have not received a bivalent COVID-19 booster dose, including the perception that additional vaccination is unnecessary because of beliefs of low booster vaccine effectiveness, misinformation about the severity of illness, or vaccination fatigue related to changes in guidance and recommendations for more doses (*9*,*10*). Access to vaccination at the facility might also have an impact.

The findings in this study are subject to at least four limitations. First, data are largely manually reported by nursing homes; therefore, misclassification of case and vaccination status of residents is possible, especially in light of changing guidance regarding what constitutes being up to date. Second, crude incidence rates and IRRs in this analysis do not account for potential nursing home or person-level confounding factors, such as time since vaccination, previous infection, age, comorbidities, community transmission rates, nursing home staff member vaccination coverage rates, or nursing home infection prevention practices. Third, the analysis did not consider residents who received a positive SARS-CoV-2 test result to be up to date with COVID-19 vaccines until 14 days after receipt of their last vaccine dose; therefore, cases among residents not up to date with COVID-19 vaccines might include infections among residents who had received a recent bivalent vaccine dose <14 days earlier. This would bias findings of difference between the two groups toward the null. Finally, the group that was not up to date comprised both unvaccinated residents and those who had received some previous vaccination but were not up to date. This precluded comparison of more specific vaccination statuses (e.g., completely unvaccinated, receipt of a complete primary series, and completed a primary series plus ≥1 monovalent booster dose) with the up-to-date group.

In this study of U.S. nursing home residents during October 2022–January 2023, differences in crude COVID-19 incidence rates among persons who were up to date with COVID-19 vaccinations and those who were not suggest that staying up to date with CDC-recommended vaccinations, which now includes receiving a bivalent booster dose, maximizes protection against COVID-19. Efforts to address barriers and increase bivalent COVID-19 booster dose coverage among nursing home residents are critical to preventing illness, severe disease, and death in this population.

**Summary**

**What is already known about this topic?**

COVID-19 vaccines are effective against SARS-CoV-2 infection in nursing home residents; however, the impact of recently recommended vaccinations, including bivalent booster doses, in this population is unknown.

**What is added by this report?**

Nursing home residents who were not up to date with recommended COVID-19 vaccines had a 30%–50% higher risk for acquiring SARS-CoV-2 infection compared with residents who were up to date with COVID-19 vaccines.

**What are the implications for public health practice?**

This study supports other recent findings that the bivalent booster dose offers additional protection in persons who previously received monovalent vaccines. Nursing home residents can maximize protection against COVID-19 by receiving bivalent COVID-19 booster doses to stay up to date with recommended COVID-19 vaccinations.

## Footnotes

*
https://www.cdc.gov/nhsn/ltc/weekly-covid-vac/index.html

†
https://www.cms.gov/files/document/qso-20-29-nh.pdf

§
https://www.federalregister.gov/documents/2021/05/13/2021-10122/medicare-and-medicaid-programs-covid-19-vaccine-requirements-for-long-term-care-ltc-facilities-and

¶
https://www.cdc.gov/nhsn/pdfs/hps/covidvax/UpToDateGuidance-508.pdf

**
https://www.cdc.gov/nhsn/ltc/weekly-covid-vac/index.html#anchor_21696

†† 45 C.F.R. part 46, 21 C.F.R. part 56; 42 U.S.C. Sect. 241(d); 5 U.S.C. Sect. 552a; 44 U.S.C. Sect. 3501 et seq.

§§ U.S. territories were excluded from the analysis because of small numbers.

¶¶
https://www.cdc.gov/nhsn/covid19/ltc-vaccination-dashboard.html
